# Synchrotron Imaging Assessment of Bone Quality

**DOI:** 10.1007/s12018-016-9223-3

**Published:** 2016-09-07

**Authors:** Shaocheng Ma, Oliver Boughton, Angelo Karunaratne, Andi Jin, Justin Cobb, Ulrich Hansen, Richard Abel

**Affiliations:** 1 Department of Mechanical Engineering, Faculty of Engineering, Imperial College London, London, SW7 2AZ UK; 2 MSk Laboratory, Department of Surgery and Cancer, Faculty of Medicine, Imperial College London, London, W6 8PR UK; 3 Department of Mechanical Engineering, Faculty of Engineering, University of Moratuwa, Moratuwa, 10400 Sri Lanka

**Keywords:** Synchrotron X-ray imaging, Microstructure, Nanomechanics

## Abstract

Bone is a complex hierarchical structure, and its principal function is to resist mechanical forces and fracture. Bone strength depends not only on the quantity of bone tissue but also on the shape and hierarchical structure. The hierarchical levels are interrelated, especially the micro-architecture, collagen and mineral components; hence, analysis of their specific roles in bone strength and stiffness is difficult. Synchrotron imaging technologies including micro-CT and small/wide angle X-ray scattering/diffraction are becoming increasingly popular for studying bone because the images can resolve deformations in the micro-architecture and collagen–mineral matrix under in situ mechanical loading. Synchrotron cannot be directly applied in vivo due to the high radiation dose but will allow researchers to carry out systematic multifaceted studies of bone ex vivo. Identifying characteristics of aging and disease will underpin future efforts to generate novel devices and interventional therapies for assessing and promoting healthy aging. With our own research work as examples, this paper introduces how synchrotron imaging technology can be used with in situ testing in bone research.

## Introduction

The remarkable mechanical properties of bone are due to the complex hierarchical structure, which is able to support the demanding loads placed on the human body during daily activities [1–6]. A better understanding of the relationship between structure and mechanical properties will enable clinicians to better recognize patients who may be more predisposed to fragility fractures, enabling preventative treatments to be initiated before they suffer debilitating fractures. With a global aging population and the increasing incidence of fractures, it is crucial that we comprehensively investigate the mechanical properties of bone in order to characterize bone health and provide sufficient information to clinicians for diagnosing fragility and improving interventions [7].

It has been challenging to characterize the mechanical properties of whole bones because the tissue has a complex hierarchical structure and multiple levels [2, 8]. Rho et al. divided the hierarchy into five length scales: the macrostructure, including trabecular and cortical bone; microstructure (10–500 µm), including osteons and single trabeculae; submicrostructure (1–10 µm) such as the lamellae; nanostructure (100–1000 nm), including mineral crystal and collagen fibrils; and subnanostructure (below a few hundred nanometers), including molecular structure [2]. Consideration of each level is required to fully understand the bulk bone material properties. As such, researchers have found it necessary to use a variety of techniques to image and test the mechanical properties at each level of the hierarchy.

Engineering testing of a specimen is usually applied to measure the mechanical properties of bone at the macrostructural level [2, 9–11]. Conventional computed tomography (CT) scans (normally known as volumetric CT) [12, 13] have been used alongside mechanical testing to map the geometry and density distribution of bone (as measured by X-ray absorption). Although the resolution is low, the smallest voxel size that can be obtained is approximately 300 × 300 × 1000 μm (pixel length × width × slice thickness) [14]. Higher-resolution benchtop micro-CT systems have been used to image bone at the microscale with voxels in the order of 5–100 µm [15, 16]. Raman spectroscopy [17–28] and Fourier transform infrared spectroscopy (FTIR) [28–31] are used to measure the chemical components of bone, such as cross-links and mineralized crystallite, at a submicron (~6.3 µm) spatial resolution [6]. At even smaller scales, transmission electron microscopy [32–35] and quantitative backscattered electron imaging (qBEI) [36–38] have been used to measure the size, shape and density distribution of mineral particles within the bone at the level of few hundred nanometers. X-ray scattering and diffraction imaging have also been used to analyze the size and shape of individual mineral crystals, including measure if thickness is in the order of 2 nm [39].

However, due to the limitations of these techniques, it has still not been possible to fully characterize bone structure. Conventional CT and micro-CT are limited to visualizing the micro-architecture of bone and are not well suited to image microdamage. Several researchers have tried staining microdamage by agitating bone samples in solutions of heavy metals such as lead and barium [40, 41]. The stain shows up well in micro-CT scans, but only the cracks that are continuous with the external surface are captured. The vascular structures are also stained which obscure the microdamage. Laser spectroscopy techniques require flat surfaces in order allow the beam to focus and are therefore limited to ground and polished bone sections [27, 42]. Grinding and polishing cause significant damage to the structure and micromechanical properties of bone tissue [42]. Transmission electron microscopy and qBEI techniques cannot combine imaging with in situ mechanical testing and also require strict sample preparation to avoid damaging the crystal in the bone before testing [35]. Similarly, conventional X-ray scattering and diffraction systems can be used to obtain static measurements. However, in situ real-time deformation testing on bone tissue cannot be performed with laboratory systems as they require long exposure time of several hours to obtain X-ray images [43].

Over the last decade, cutting-edge synchrotron imaging techniques have been developed to overcome the limitations in spatial resolution [2, 44] while facilitating nondestructive analysis. A synchrotron is a large ring (0.56 km in diameter) consisting of three components: a linear accelerator, a booster and a storage ring. Synchrotron accelerates electrons close to the speed of light and then slows them down with electromagnets; the lost energy is released in the form of high-energy monochromatic X-rays [45, 46]. This method for creating X-ray beams is the key to success of synchrotron because high-energy monochromatic beams produce less noisy images [6, 47]. The X-ray beams have been widely applied by biomedical and biomaterial scientists to investigate tissues down to the molecular level [48–51].

The high-resolution images obtained from synchrotron facilities could finally enable scientists to understand the complex relationship between structural and mechanical properties of bone. The quality of bone material and the mechanical performance of bone as a whole are ultimately dependent upon the mechanical behavior at the micro- and nanoscale [52, 53]. Therefore, a better understanding of bone micro- and nanostructure would be useful for understanding bone health and disease. However, the nano- and microscale mechanics of bone are poorly understood [2]. Synchrotron imaging techniques could be combined with in situ mechanical testing to visualize the structure and mechanical behavior of bone at these levels.

Indeed, many researchers have already carried out innovative research using synchrotron [52, 54–58]. Larrue et al. applied synchrotron micro-CT to image bone microdamage in human trabecular bone specimens and characterize the size and shape in 3D [55]. In this study, the authors compared the use of synchrotron micro-CT to histological techniques that have been the predominant approach in investigating bone quality. Synchrotron technology revealed that microdamage morphology appears to be more complex in 3D compared to 2D. Moreover, 3D analysis reduces ambiguities of microcrack morphology present with 2-D analysis [55]. Researchers using synchrotron SAXS and WAXD imaging have demonstrated the utility for the assessment of bone nanostructure, in particular, the arrangement and orientation of mineral platelets and collagen fibrils in bone [52, 54]. Thus, the potential of synchrotron technology in advancing research in bone quality is becoming increasingly clear.

The aim of this paper is to review and explain the use of novel synchrotron imaging techniques for visualizing bone structure and mechanics at both the micro- and nanoscale, using examples from our own work to explain how the data were captured and analyzed. The paper will give scientists and clinicians a broad overview of the applications of synchrotron imaging techniques in bone research, so that they might be able to apply such techniques in their own research into aging, disease or surgical interventions. There are three key techniques, which will be discussed: micro-CT, SAXS and WAXD.

## Synchrotron Imaging Technology

### Microscale X-ray Tomography

Synchrotron can image the internal properties of bone with a higher spatial resolution and intensity than laboratory-based techniques because the system can fully exploit phase contrast [59, 60]. Phase contrast takes advantage of the fact that bone, marrow and air have different refractive indices, which produces a shift in direction of the X-rays passing through the sample, particularly when the X-rays pass the boundary between the tissues. The technique is particularly useful for enhancing the contrast of surfaces and interfaces in samples, which would not be visible using conventional absorption CT. Although phase-contrast scanning can be accomplished with laboratory setups (e.g., GE Nanotom, GE, USA), it is possible to achieve higher spatial resolution with synchrotron instrumentation. This is because the synchrotron X-ray beam is monochromatic rather than polychromatic (i.e., the X-rays have a single energy, and the resulting images are less noisy) and also because the distance from the specimen to the X-ray detector panel can be several meters (the diffraction is easier to measure at large distances). This is an important advantage because the resolution of the synchrotron scans is higher, even though the Nanotom system can image larger objects at smaller voxel size. Voxels from the synchrotron system vary from about 1.3 to 19.1 µm depending on the field of view (i.e., maximum sample diameter) which ranges from 3.3 to 48.8 mm [61], whereas voxels in the Nanotom system range from 0.3 to 80 µm and sample diameter from ~1 to 240 mm [62].

**Fig. 1:**
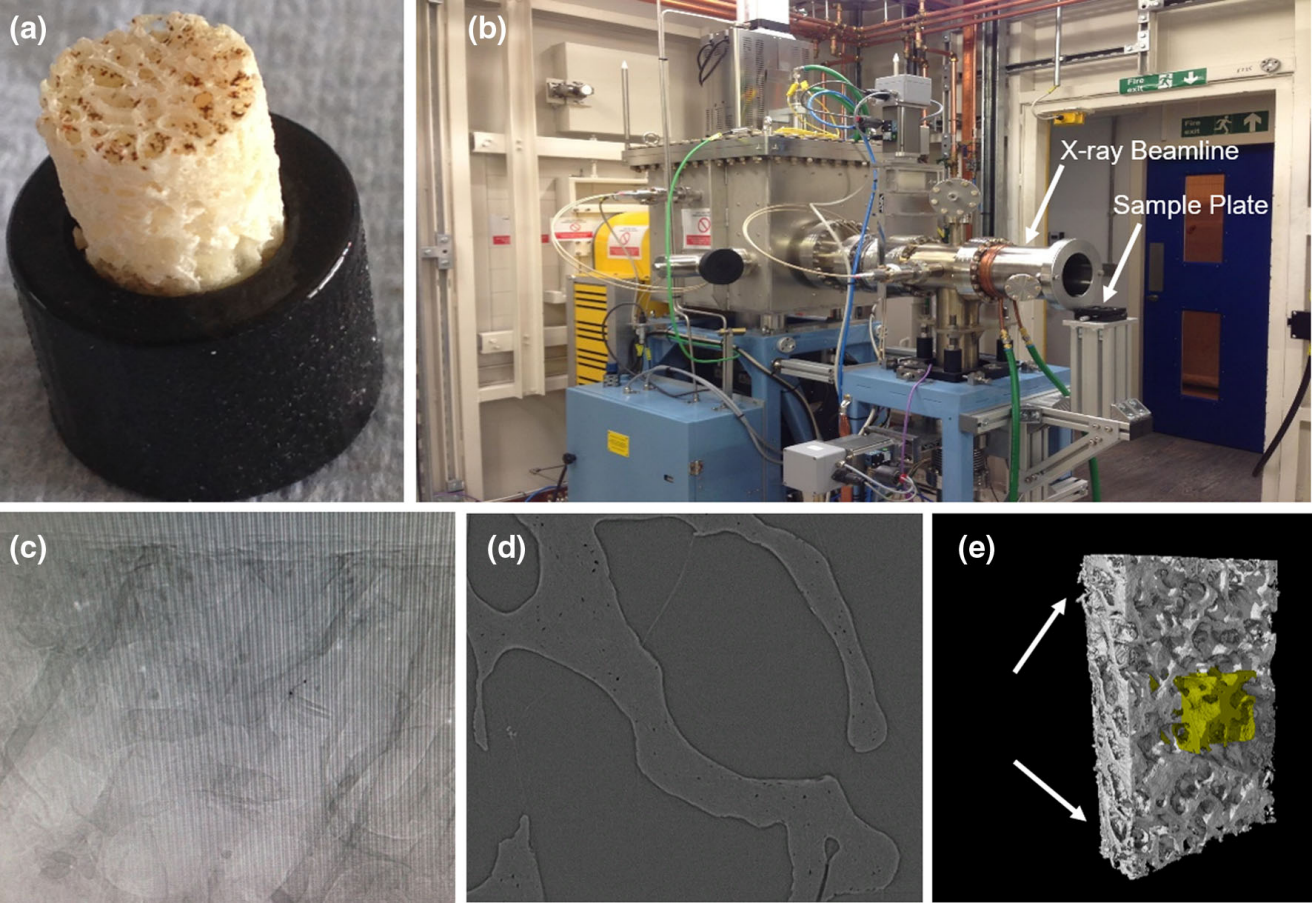
Synchrotron micro-CT scan of a trabecular core. **a** trabecular core mounted into a 3D-printed holder. **b** X-ray source. **c** Raw tomography projections. **d** Reconstructed CT-slice with 1.3 μm/voxels. **e** 3D model of entire bone core with artifactual drilling damaged on the edge (*white arrow*) and a synchrotron-scanned region shown in *yellow* which is far away from the drilling zone

**Fig. 2:**
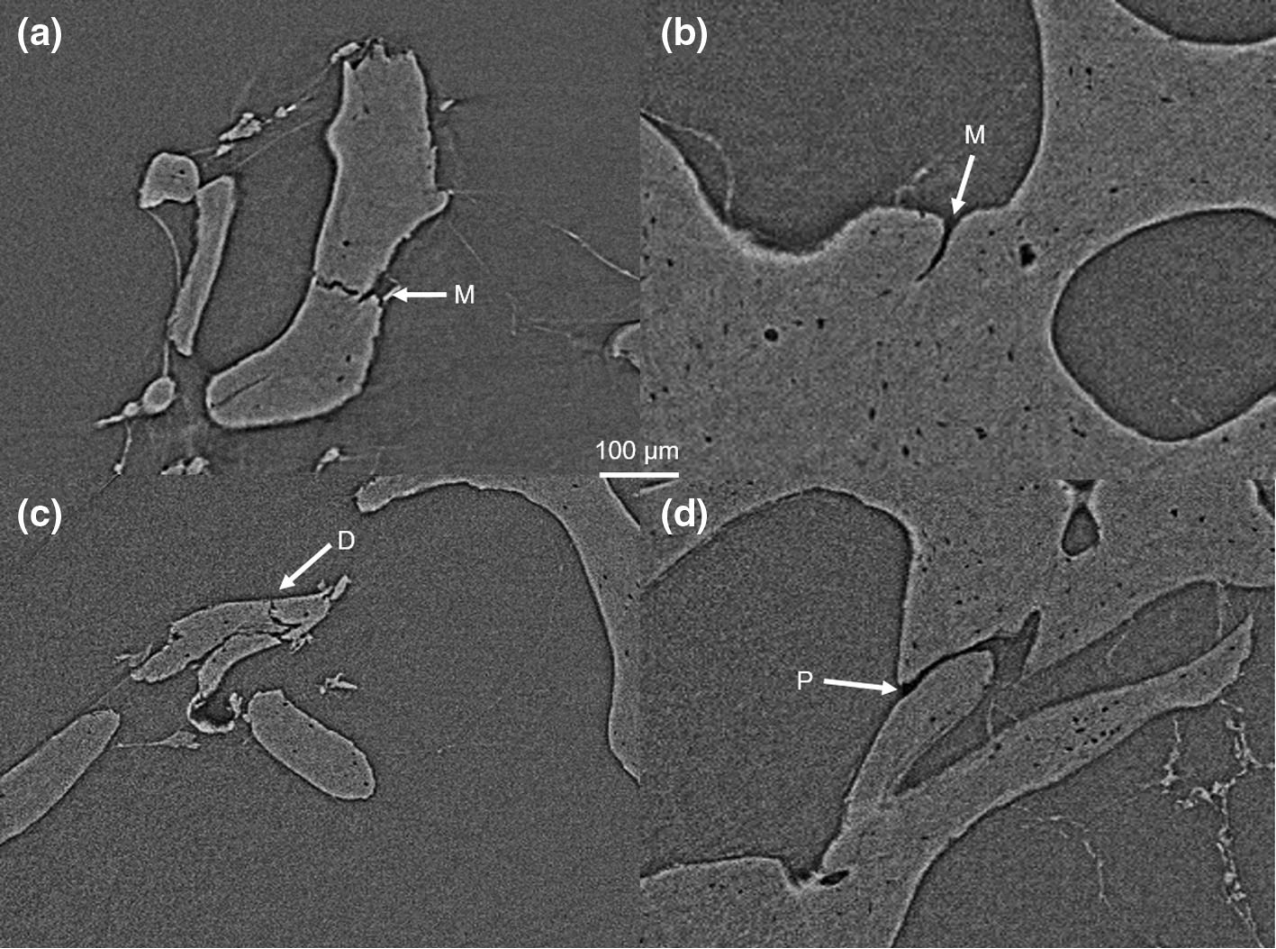
Synchrotron micro-CT visualizes microcracks (*M*), diffuse damage (*d*) and perforations (*P*) within trabeculae. The *white* and *black lines* at the edge of the bone are a beam-hardening artifact that were intensified by the phase-contrast imaging process which detects X-ray refraction

An example experiment for imaging trabecular micro-architecture is presented in Fig. 1. A bone specimen (Fig. 1a, 7 mm in diameter and 10 mm in length) was drilled out from a femoral head then mounted into a 3D-printed holder. The holder was then attached onto the sample plate in the synchrotron scanner (Fig. 1b). Sample preparation and mounting can damage the outer 1 mm of bone. To avoid scanning damaged bone tissue, it was necessary to image a volume of interest within the bone core which was 3.2 mm in diameter (Fig. 1e). The sample was scanned using an X-ray beam with 3.1281 J ring energy and 301.5 mA ring current. The imaged volume was 3.28 × 3.28 × 2.76 mm with a voxel size of 1.3 µm. The system collected a radial stack of raw projection data (i.e., shadow grams) at 6400 angles which were 0.056° apart (Fig. 1c). The radial stack was converted into a longitudinal stack of 2000 micro-CT slices by applying reconstruction algorithms [63] to the projection data (Fig. 1d). The CT slices can be used for microstructural and micromaterial analysis of the trabecular bone down to ~1.3 µm voxel size. At this level, microcracks [64], diffuse damage [65] and perforations [66] can be clearly visualized, counted and measured (Fig. 2). Synchrotron micro-CT is the only suitable nondestructive 3D imaging technique for visualizing and quantifying microdamage of bone in high resolution without any contrast agents. Standard micro-CT scanners cannot achieve sufficient resolution, even with contrast agents. Confocal microscopy can be used to produce 3D images of microdamage but only at a depth of about 200 µm.

**Fig. 3:**
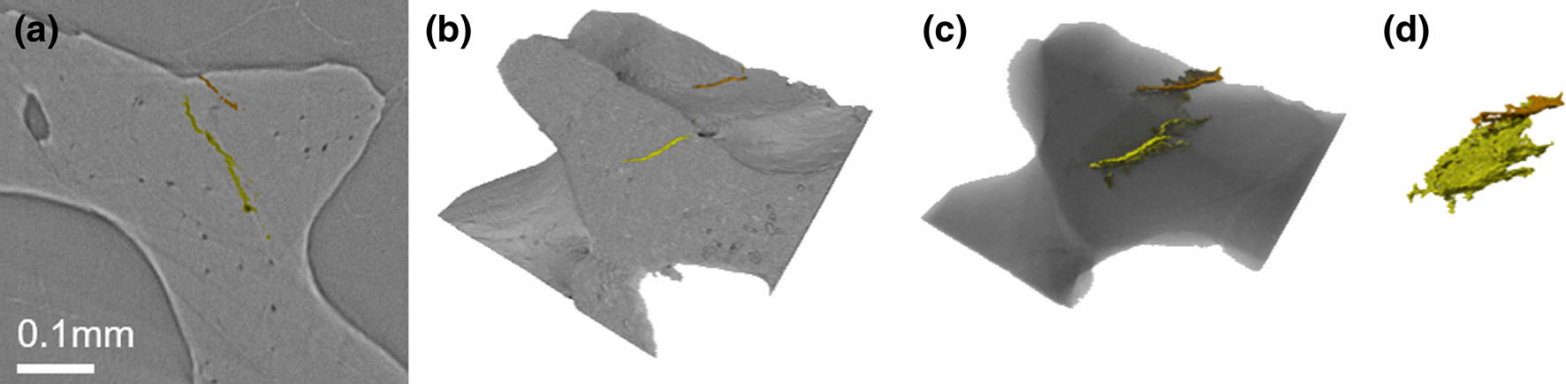
Trabecular bone microcracks. **a** Slice from synchrotron micro-CT scan depiciting microcracks. **b** 3D reconstruction of microcracks at the bone surface and **c** a transparency revealing the path inside the bone. **d** Rendered image of the crack surface

Synchrotron micro-CT slices can be reconstructed into 3D computer models using image processing software such as Mimics (Leuven, Belgium), Avizo (Hillsboro, USA) and VGStudio MAX (Heidelberg, Germany). 3D reconstructions can also be used for quantitative assessment of microdamage, which might be able to provide an intuitionistic view of microdamage in bone. The advantages of using 3D reconstructions to measure the amount of microdamage in bone are that the 3D model prevents over-counting or undercounting of microdamage that may occur when assessing microdamage using 2D slices. In Fig. 3, it can be seen that two microcracks could be counted as separate in the 2D version, but are clearly merged after reconstructing the 3D structure. The 3D models can also be used to measure the dimensions such as volume and length of microcracks [55].

Traditionally, histological sections have been used to visualize microcracks [67]. Histology presents a number of problems when counting and measuring microcracks. First, it is difficult to identify cracks that are orthogonal to the slice plane. Secondly, it is difficult to measure the entire length of a crack that crosses several slices [68–70]. As such histologists tend to only measure ‘crack length’ in a single slice, thereby underestimating the length [70]. Furthermore, it is not possible to combine structural analysis with mechanical testing because both techniques are destructive.

Another advantage of phase-contrast synchrotron micro-CT is that the technique can be combined with in situ mechanical testing to investigate structural deformations and mechanical behavior under load [71–75]. Synchrotron micro-CT even enables visualization of microcrack growth inside bone material, within the context of the microstructure. Micro-CT images can be collected in the static state, and then under stages of loading until the sample fails. Afterward, the imaging stacks can be processed using finite element model analysis (FEA) [76–78] to map stress and strain distribution and digital volume correlation (DVC) [79–81] to measure crack propagation. The recorded loads and displacements from mechanical testing can be used to calculate material properties, such as the Young’s modulus, crack-opening displacement and fracture toughness. At the same time, propagation of microcracks and diffuse damage in the specimen can be tracked during each mechanical loading step. Accurate measurements of bone nano- and micromechanics could potentially be combined with whole bone imaging and FEA to analyze the contribution of the collagen–mineral matrix to whole bone mechanical properties [82].

### Nanoscale X-ray Imaging

X-ray electrons and photons have very small wavelengths (~0.1 nm) and scatter or diffract off nanoscale objects [83]. Therefore, synchrotron SAXS and WAXD spectra (i.e., scattering and diffraction patterns) can be used to resolve the structural properties of bone mineral platelets and collagen fibrils at the nanoscale, which are important for understanding the mechanical properties of the entire bone [84]. By combining in situ micromechanical testing and synchrotron SAXS and WAXD techniques, the behavior of the mineral particles and collagen fibrils under load can be tracked [52, 56, 57, 84, 85]. Using SAXS and WAXD spectra, it is therefore possible to directly resolve the ultrastructural strains of fibril and mineral platelets and determine the orientation of mineral platelets and fibrils within bone lamella.

Synchrotron SAXA and WAXD methods are superior to conventional techniques because the high X-ray intensity allows for real-time acquisition of spectra (as opposed to hours) [86]. Further, synchrotron machines use modern area detectors (large active areas and small pixel sizes) which, in combination with high-brilliance source (high photon flux and small divergence), allows the detector to be placed much closer to the sample without compromising spatial resolution. This allows compact experimental setups to obtain SAXS and WAXD signals which can even be measured simultaneously with the same detector, covering the wide range of scattering angles [87].

For measuring SAXS spectra during deformation, the sample to detector distance is quite long (1–3 m to provide a small angle), while for the WAXD spectra, the distance between the sample and detector is shorter (0.3 m to provide a wide angle). Additionally, the thickness of the testing specimen should be <1 mm to allow penetration of X-rays. Optimal thickness of the bone section can be determined using the relationship between the X-ray scattering intensity, thickness of the specimens and the linear absorption coefficient as described elsewhere [52, 54, 58, 88]. The SAXD/WAXD spectra can be analyzed to extract the nanomechanical parameters, such as fibril strain, mineral strain, by using the CAKE or INTEGRATE command in the software FIT2D (Hammersley, Grenoble, France) [89].

**Fig. 4:**
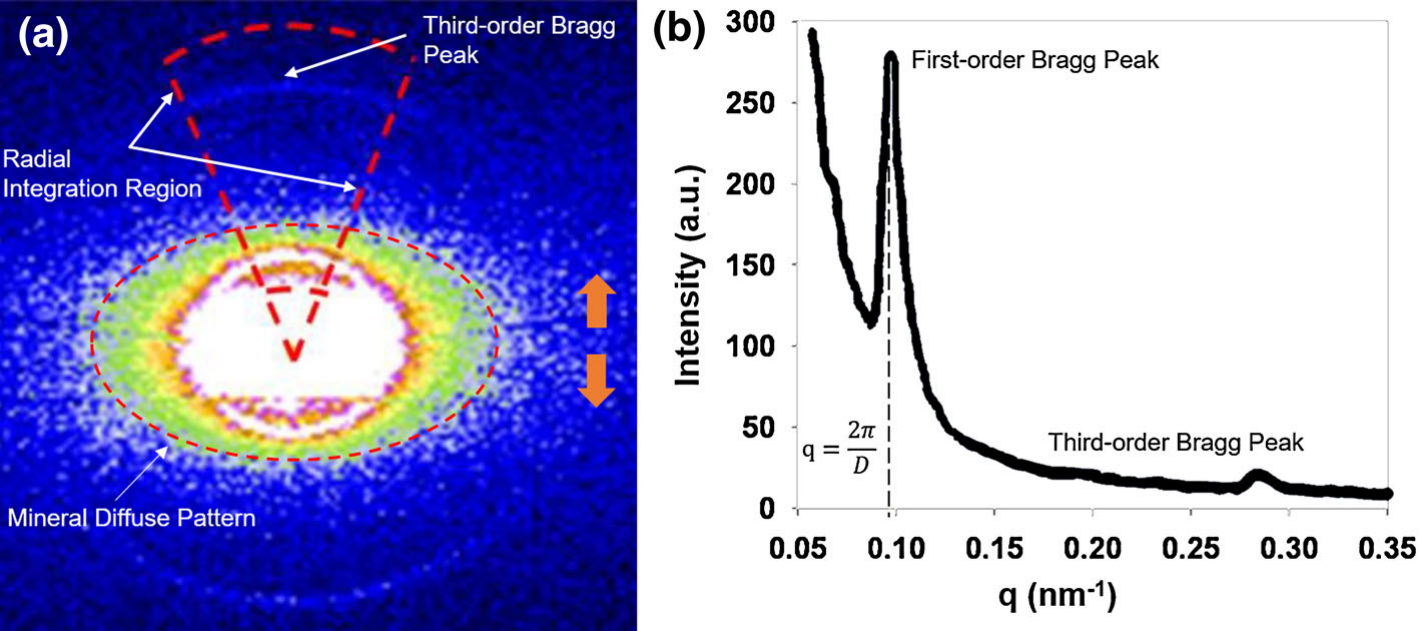
Typical example of SAXS spectra from bone material, **a** *Dashed elliptical* sector denotes region of diffuse scatter in SAXS spectra from mineral platelets. Dashec CAKE sector is the radial integration region in q-space command from FIT2D for the third-order Bragg peak shown by the *blue arrow*. The *orange arrows* represent the loading axis, **b** Shows a 1D plot from radial integration in (**a**). Both first and third diffraction peaks resulting from the collagen meridional D-periodicity can be seen in the azimuthally 1D plot. The third order is the best integral peak because the first-order peak has a much higher diffuse noise from mineral platelets

Typical SAXS spectra from mineralized collagen fibrils of bone under load are shown in Fig. 4 (the orange arrow displays the loading axis in Fig. 4a). SAXS bone spectra normally contain two distinct parts: the diffuse scatter from the mineral platelets (the ellipse region in center of Fig. 4a) and a group of Bragg diffraction peaks (blue rings in Fig. 4a). The shape and the elongated direction (Fig. 4a) in SAXS spectra can be used to measure the degree of orientation of the mineral platelets with respect to the collagen fibrils. According to Schematic Ewald sphere theory [90], the plane of the collagen fibrils needs to be perpendicular to the X-ray beam and parallel to the loading axis [58]. Otherwise, the information on collagen fibril mechanics cannot be detected and analyzed correctly. For more detailed information about the orientation of mineral platelets in SAXS spectra, please refer to the paper by Fratzl et al. [6]. The synchrotron scans can be collected in combination with stepwise in situ mechanical testing, and the SAXS scans can be successively measured at each loading stage. Then, the shift of Bragg peaks [91] in the stepwise loaded diffraction pattern can be compared with the reference scan at zero stress in order to measure the collagen fibril strain.

The Bragg peak is a pronounced peak on the Bragg curve which plots the energy loss of ionizing radiation during its travel through bone. The Bragg curve is obtained using the SAXS spectral image. Generally, the third-order Bragg peak (Fig. 4a) is used for radial integration [58] to measure the shift during deformation because the first order is obscured by noise from the mineral diffuse scattering pattern in the center. The angular distribution of SAXS intensity values is radially integrated to a 1D plot (Fig. 4b). A series of 1D plots from radial integration can be used to measure the peak shift, which can be converted to calculate fibril strain. The SAXS spectra (diffraction pattern) might only be able to measure the average fibril strain parallel to the loading axis because it would be difficult to separate the collagen elongation and shearing components [58].

**Fig. 5:**
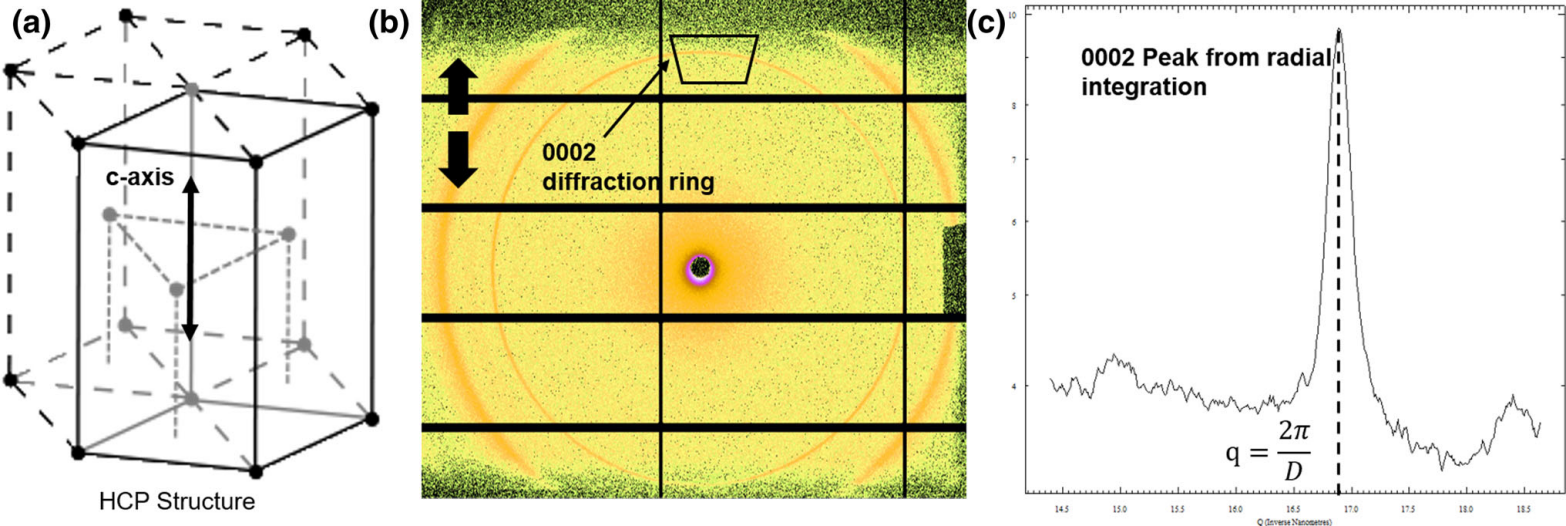
A typical example of WAXD spectra from bone material **a** a HCP structure of mineral platelet showing C-axis. **b** The *bold black arrows* represent the loading axis, and the *black arrow* indicate the 0002 diffraction ring to track tie c-axis in HCP structure. A *black trapezium* sector is used for a radial integration to obtain the 1D peak plot of 0002 diffraction ring. The *black rectangular* lattice on the detector can be neglected as they are regions used for measurements between active Cefaclor areas on the Plitatus P3-2M detector, **c** the azimuthally 1(q) 1D plot from the 2D trapezium section in (**b**)

WAXD is also used to investigate the nanoscale structure and mechanics of bone (Fig. 5). Bone specimens are loaded in a stepwise manner with WAXD spectra collected in series at each loading stage. The strongest diffraction occurs at the central (c) axis of the crystalline hydroxyapatite of a mineral platelet [92, 93] which has a hexagonal closed-packed (HCP) structure (Fig. 5a). The diffraction pattern appears as an orange ring in the WAXD spectral image (Fig. 5b). The ring is referred to as the ‘0002’ lattice diffraction ring. Under loading, the mineral platelets deform resulting in a shift of the 0002 diffraction pattern. In the example Fig. 5., the c-axis of the mineral platelets is parallel to the loading axis (black arrow in Fig. 5a) which means the shift in 0002 diffraction peak (Fig. 5b), when compared to the reference at zero stress, can be used to measure mineral strain along the c-axis. The shift in the 0002 diffraction peak is measured from the radial integration of the trapezoidal region around the 0002 ring, which produces a 1D plot (Fig. 5c) where the shift is described by the width of the 0002 peak.

## Translating Synchrotron into Clinical Practice

Synchrotron-computed micro-CT provides detailed information about the 3D bone structure, including porosity [71, 94, 95]. Dual-energy X-ray absorptiometry (DXA) is currently used in clinical practice to measure the bone mineral density (BMD) and predict fracture risk of patients. Yet, the technique does not fully explain the increase in fracture risk with age because the images are 2D and do not capture the complex hierarchical structure of bone and the variation in tissue-level mechanical properties of bone [14, 96]. Synchrotron micro-CT is occasionally used for accurately measuring tissue mineral density [97] and also can be used to analyze the porosity and other structural features of bone, such as microcracks, [71]. Bone is an anisotropic material, meaning that stiffness properties vary from one direction to another [98]. Synchrotron SAXS/WAXD spectra can provide information about the orientation and nanomechanics of the mineral platelets and the collagen fibrils, partly explaining why the elastic properties of bone vary from one direction to another [95, 99, 100].

Synchrotron-sourced techniques cannot currently be used in vivo due to the high radiation dose, but the images are enabling clinicians and scientists to understand how and why healthy and aging or diseased bone behaves differently under load. To be able to predict how a bone behaves under load, it is important to know the loads on the bone (directions and magnitudes), the structure of the bone in 3D and the mechanical properties of the bone at different length scales [101]. Fracture properties are very important at all length scales as the mineralized collagen fibril fracture properties influence the whole bone fracture properties. By understanding the mechanical properties of a patient’s bone, clinicians may be better able to predict fracture risk and predict treatment outcomes. The stiffness properties of bone are particularly of interest at the apparent level or meso-millimeter scale as it is at this level that orthopedic implants and devices may interact with the bone, e.g., fixation and stress shielding.

### Assessment of Bone Health at the Point of Care

By combining high-resolution imaging techniques such as synchrotron-computed micro-CT with material testing techniques such as tensile and 3 or 4 point bending and micro- or nanoindentation, we may better model bone fracture properties in vivo and apply this knowledge to clinical practice. For example, synchrotron techniques could be used to test the usefulness of novel in vivo devices. Hengsberger et al. combined nanoindentation [101] testing with synchrotron-computed microtomography to predict the apparent Young’s modulus (stiffness measured at the millimeter scale level per unit volume) of cortical bone [71]. The apparent modulus was determined using traditional tensile mechanical testing. The same bone underwent synchrotron CT scanning and then nanoindentation. Although the authors only tested three specimens, the results showed that that the apparent modulus values calculated by nanoindentation combined with porosity information from synchrotron CT were similar to the apparent modulus values from tensile mechanical testing of bone at the apparent level [71]. Therefore, synchrotron micro-CT is useful for determining the apparent Young’s modulus of bone when combined with nanoindentation elastic modulus information.

Akhtar et al. [100] employed synchrotron WAXD combined with nanoindentation to investigate the role of apatite crystals in trabecular bone under loading. They performed uniaxial compression tests of the bone while undergoing synchrotron WAXD scanning. The authors reported that the apatite crystals were aligned in the same direction as the trabeculae and that the trabeculae and apatite crystals running parallel to the direction of loading experienced the most strain during loading, demonstrating the anisotropy of bone. This study used antler bone, so they used nanoindentation to characterize the elastic modulus of the bone prior to the synchrotron WAXD compression experiments [100].

These studies demonstrate that synchrotron imaging technology is a powerful tool, enabling clinicians, scientists and engineering researchers to investigate and understand the fracture and elastic properties of bone at the micro and nanoscale. Synchrotron imaging studies can be used to investigate how healthy and diseased bones differ in both structure and mechanical properties at this scale. This could lead to better design and monitoring of medications to treat conditions of the bone, such as osteoporosis, as well as optimizing orthopedic implants and devices to closer match patients’ bones, improving implant survival and reducing fractures of the bone around implants.

### Limitations of Synchrotron Imaging

There are many factors that prevent synchrotron imaging technology being applied in clinical practice and limit the extent to which systems can be used in preclinical or clinical research. The cost of building and maintaining a particle accelerator ring is very high. For example, the facility we used at The Diamond Light Source (Didcot, UK) cost ~$650 million to build and costs >$52 million to run annually [102]. Accelerators must be located in high-security zones approved for high-radiation sources and can take a decade to build.

**Fig. 6:**
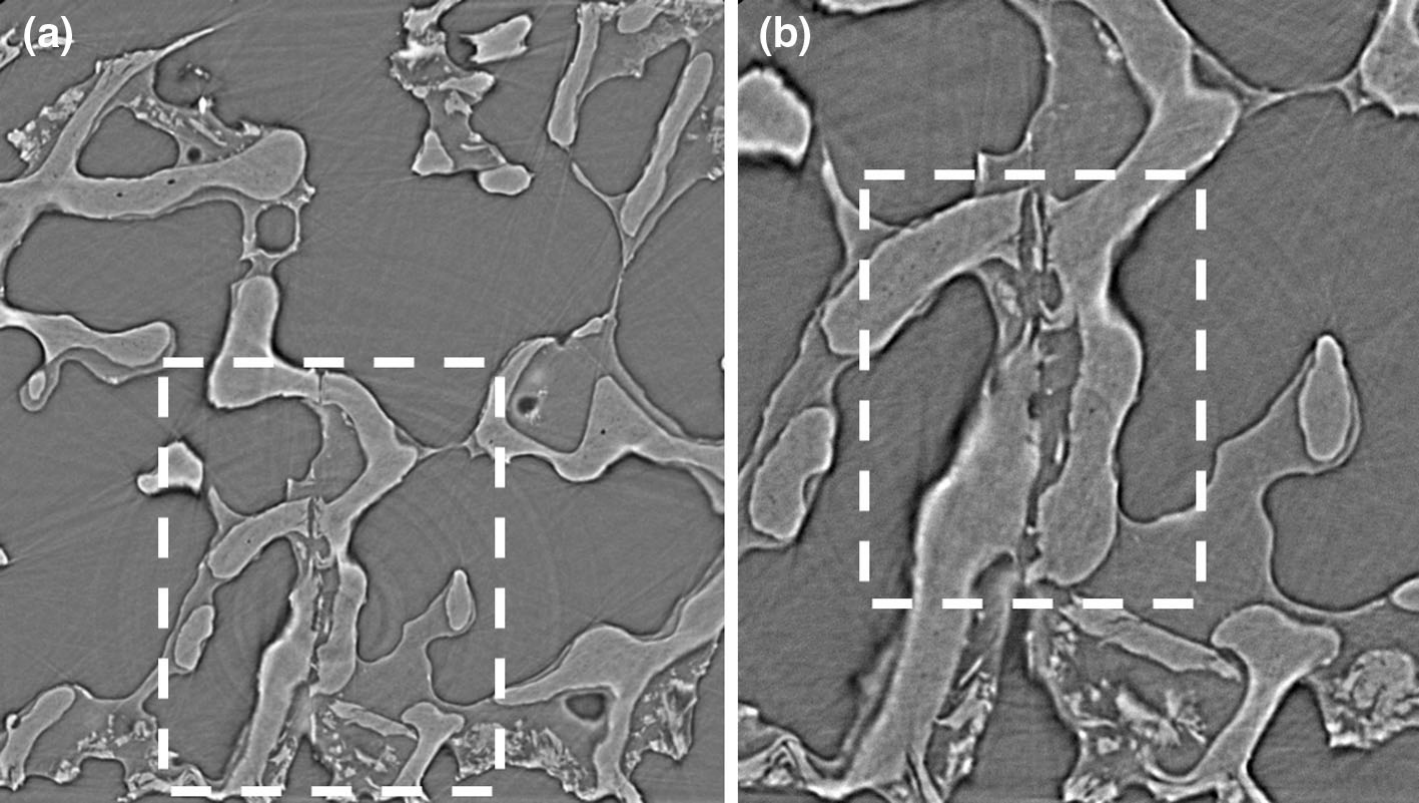
Two different spatial resolution scans from I12 beamline at Diamond Light Source, **a** module 3 scan (3.2 μm/voxel) can only show a general view of the trabecular structural at the same position with precracked three-point bending experiment, **b** module 4 scan (1.28 μm/voxel) clearly shows a precracked on the bone sample, while *dashed sector* in (**a**) can be dearly visualized in (**b**). However, module 4 takes 60 min per stack, and it is not recommended here as the sample will experience long-time exposure at each loading stage, in which the radiation damage will change the chemical component of the bone and affect the mechanical properties of the experiment

The high-energy X-ray beams that allow synchrotron to capture high-resolution data also cause more radiation damage than laboratory or hospital CT instruments, especially in biomaterials under mechanical testing. Figure 6 shows two micro-CT slices from two different modules (with different spatial resolutions) from the I12 beamline at the Diamond Light Source. The module 3 slice (3.2 µm/voxel in Fig. 6a) has a lower spatial resolution than the module 4 slice (1.28 µm/voxel in Fig. 6b), and therefore, lower contrast and blurred boundaries can be observed. However, collecting a module 4 micro-CT stack takes ~60 min, while module 3 only requires ~2 min. As a result, much less radiation damage will occur using module 3 to image bone specimens, and therefore, module 3 is recommended when combined with mechanical testing. Even though module 4 was able to provide a much higher-resolution image of the bone, it cannot be recommended as optimal because the long radiation exposure time during each loading stage could significantly damage the collagen matrix inside of bone, potentially reducing the mechanical properties, such as strength, ductility and toughness [74]. It is important to notice that for bone material, a safe level is between 30 and 35 kGy [75], and material properties would be significantly changed above 70 kGy [74].

In addition, synchrotron experiments take a long period of time to prepare for, up to 1 year. This is due to the time taken to apply for time on the beamline (via a competitive application process), custom designing and building a specific testing rig and bone sample preparation. Any experimental team will absolutely require at least one synchrotron expert who can help to design and run the experiment, especially during the beam time when alternative experimental plans need to be developed if (as is nearly always the case) the original experimental design does not work. This will prevent the team from wasting the valuable allocated beam time.

## Conclusion

Synchrotron imaging allows researchers to view the nano- and microstructure of bone at incredibly high spatial resolution, while simultaneously measuring the mechanical properties of the bone. The data will be used to elucidate the relationship between bone hierarchical structure and mechanical properties, particularly at the micro- and nanoscale. Ultimately, the knowledge acquired will help clinicians to better understand the aging process and pathophysiology of bone fragility. Although synchrotron cannot be directly applied in vivo due to its high radiation dose, the technology will help clinicians to develop novel diagnostic tests for disease, identify novel treatment targets and improve interventional outcomes thereby improving patient care and quality of life.
